# Chi-miR-3031 regulates beta-casein via the PI3K/AKT-mTOR signaling pathway in goat mammary epithelial cells (GMECs)

**DOI:** 10.1186/s12917-018-1695-6

**Published:** 2018-11-27

**Authors:** Kaiwen Chen, Jinxing Hou, Yuxuan Song, Xiaochuan Zhang, Yuhan Liu, Gonghai Zhang, Kai Wen, Haidong Ma, Guang Li, Binyun Cao, Xiaopeng An

**Affiliations:** 1 College of Animal Science and Technology, Northwest A&F University, No. 22 Xinong Road, Yangling, Shaanxi 712100 People’s Republic of China; 2 Animal Engineering Branch, Yangling Vocational & Technical College, No. 10 Xinong Road, Yangling, Shaanxi 712100 People’s Republic of China

**Keywords:** Dairy goats, miRNAs, Gene expression, Transcriptome, Milk components

## Abstract

**Background:**

MicroRNAs can regulate gene expression at the posttranscriptional level through translational repression or target degradation. Our previous investigations examined the differential expression levels of chi-miR-3031 in caprine mammary gland tissues in colostrum and common milk stages.

**Results:**

The present study detected the role of chi-miR-3031 in the lactation mechanisms of GMECs. High-throughput sequencing was used to analyze transcriptomic landscapes of GMECs transfected with chi-miR-3031 mimics (MC) and a mimic negative control (NC). In the MC and NC groups, we acquired 39,793,503 and 36,531,517 uniquely mapped reads, respectively, accounting for 85.85 and 81.66% of total reads. In the MC group, 180 differentially expressed unigenes were downregulated, whereas 157 unigenes were upregulated. KEGG pathway analyses showed that the prolactin, TNF and ErbB signaling pathways, including *TGFα*, *PIK3R3*, *IGF2*, *ELF5*, *IGFBP5* and *LHβ* genes, played important roles in mammary development and milk secretion. Results from transcriptome sequencing, real-time PCR and western blotting showed that chi-miR-3031 suppressed the expression of IGFBP5 mRNA and protein. The expression levels of β-casein significantly increased in the MC and siRNA-IGFBP5 groups. We observed that the down-regulation of IGFBP5 activated mTOR at the Ser2448 site in GMECs transfected with MC and siRNA-IGFBP5. Previous findings and our results showed that chi-miR-3031 activated the PI3K-AKT-mTOR pathway and increased β-casein expression by down-regulating IGFBP5.

**Conclusions:**

These findings will afford valuable information for improving milk quality and contribute the development of potential methods for amending lactation performance.

**Electronic supplementary material:**

The online version of this article (10.1186/s12917-018-1695-6) contains supplementary material, which is available to authorized users.

## Background

Previous studies have shown that mammary epithelial cells (MECs) play key roles in milk protein and lipid biosynthesis [1–3]. During lactation, MECs secrete large quantities of milk proteins, notably caseins. Caseins (αS1-, αS2-, β-, and κ-CN in most species) are the main milk-specific proteins [4]. Transcriptomic regulations affect the changes of milk composition and yield in MECs. Thus, in this study, MECs were harvested from the mammary gland.

RNA sequencing (RNA-seq) provides a holistic view of transcriptomes, allows the refinement of gene structures and also uncovers novel splice isoforms, SNPs and transcribed regions [5–7], in addition, it also produces complete gene expression measurements instead of relative measurements, thereby providing more significant insights and accuracy than microarrays [8, 9]. Using a transcriptome analysis, Suarez-Vega et al. (2017) found that RNA transport, and fatty acid elongation Jak-STAT signaling pathways could affect milk yield, protein percentage and fat percentage [10]. Via an RNA-seq analysis of mammary epithelial cells, Wanyonyi et al. (2017) suggested that seven pathways, including lysosome, galactose metabolism, p53 signaling, cell adhesion molecule (CAM), complement, coagulation and Nod-like receptor signaling pathways, are only significantly responsive to extracellular matrices in the presence of lactogenic hormones [11].

MiRNAs are noncoding small RNA molecules (about 22 nucleotides) that regulate gene expression post-transcriptionally by binding to the 3′-UTR of their target mRNAs [12, 13], which refer to proliferation [14], apoptosis [15], reproduction [16] and milk performance [17, 18]. The overexpression of miR-130a notably decreases the levels of cellular triacylglycerol (TAG) and suppresses lipid droplet formation, but the inhibition of miR-130a results in increased TAG accumulation and lipid droplet formation in bovine mammary epithelial cells [19]. The miRNA target network shows that bta-miR-574 can affect lactation and mammary gland development by leptin receptor, which is involved in the adipocytokine signaling pathway. MiR-3880 regulates the development of mammary gland and lactation by serine/threonine-protein phosphatase, which participates in the oxytocin signaling pathway [20]. The miR-15b expression decreases during lactation and oppositely correlates with lipid synthesis proteins, suggesting that miR-15b may refer to milk production and lipid synthesis [21]. The overexpression of miR-145 notably increases TAG accumulation, fat droplet formation, and proportions of unsaturated fatty acids. Oppositely, silencing miR-145 impairs fatty acid synthesis [22].

The insulin-like growth factor (IGF) system mainly includes IGF-I and IGF-II ligands, IGF-IR and IGF-IIR receptors and a family of IGF-binding proteins (IGFBPs) in vertebrates [23]. This system performs indispensable roles in adjusting growth, development and reproduction [24]. The functions of IGFs are regulated by their receptors and IGF-IGFBPs that serve as major regulators of IGF activity through acting as carriers [25]. These molecules also dominate the bioactivity and bioavailability of IGFs that interact with IGF receptors to modulate downstream signal transduction networks [26]. IGFBP5 is more conserved than other IGFBPs in mammals [27, 28]. A further study showed that IGFs act as important upstream activators of PI3K/AKT-mTOR signaling pathways to regulate milk protein synthesis [29–31]. The effect of milking frequency on milk yield is locally controlled within mammary glands and can be a function of changes in either the number or activity of secretory mammary epithelial cells. IGF-I signaling can mediate these effects because it can be locally controlled within mammary glands [32].

Our previous study examined the differential expression levels of chi-miR-3031 in caprine mammary gland tissues in colostrum (2 days postpartum) and common (90 days postpartum) milk stages [20]. Bioinformatics analysis suggested chi-miR-3031 and IGFBP5 are key signaling factors that regulate protein synthesis and cell proliferation in GMECs. IGFBP5 can regulate IGFs downstream of signal transduction networks by interacting with IGF receptors [26–29]. Until now, there have been no reports on the effects of chi-miR-3031 and IGFBP5 on milk protein synthesis. Therefore, in the present study, we used chi-miR-3031 mimics and mimic negative controls to transfect GMECs. We performed RNA-Seq to identify DEGs and the relative signal pathways affecting protein synthesis in GMECs. Among the identified DEGs, due to their involvement in lactation mechanisms, we selected IGFBP5 based on bioinformatics for further studies. This study aimed to determine the regulatory mechanisms of chi-miR-3031 on casein synthesis in GMECs.

## Results

### Summary of transcriptome sequencing

RNA-Seq was used to analyze the transcriptomic landscapes of GMECs transfected with MC and NC. Total RNAs from GMECs were extracted to construct RNA libraries for Illumina sequencing. Reads with adaptors and low-quality reads were removed prior to assembly. In total, we acquired 46,351,307 and 44,748,620 clean reads from each library (Table 1). In the MC and NC groups, uniquely mapped reads totaled 39,793,503 and 36,531,517, respectively, accounting for 85.85 and 81.66% of total reads. Only the uniquely mapped reads were considered in this analysis. Distribution of reads in different regions of the reference genome was evaluated using the following standard metrics: exon, intron and intergenic reads. Table 2 summarizes these results.

**Table 1:** Summary of sequence read alignments to the reference genome

Category	Chi-miR-3031		Negative control	
	Reads number	Percentage	Reads number	Percentage
Total reads	46,351,307	100%	44,748,620	100%
Total mapped reads	43,734,071	94.35%	40,333,973	90.16%
Multiple mapped reads	3,940,568	8.50%	3,802,457	8.50%
Uniquely mapped reads	39,793,503	85.85%	36,531,517	81.66%
Reads mapped in proper pairs	37,712,533	81.36%	33,479,622	74.82%

Note: Total reads: total number of sequencing reads. Total mapped reads: the reads that aligned to reference sequence and its ratio. Multiple mapped reads: in total mapped reads, reads aligned to two or more places. Uniquely mapped reads: in total mapped reads, reads aligned to only one position. Reads mapped in proper pairs: paired-end sequencing sequence aligned to the chromosome reasonable direction and position

**Table 2:** The distribution of reads in the goat genome in different areas

Category	Chi-miR-3031	Negative control
Exon reads	85.53%	85.93%
Intron reads	7.36%	6.95%
Intergenic reads	7.11%	7.12%

### Identification of DEGs

Although a number of genes were differentially expressed between the two libraries, the present study focused on genes meeting the designated criteria of *p* values < 0.05 and |log_2_ FoldChange| > 1 (Additional file 1: Table S1). According to changes in relative gene abundance between the two libraries, 180 differentially expressed unigenes were down-regulated, and 157 unigenes were up-regulated in the MC group compared with the NC group of GMECs (Additional file 1: Table S1). Differential expression of *transglutaminase 3*, *thromboxane synthase*, *ATPase, H+ transporting*, *lysosomal accessory protein 1-like*, *fragile histidine triad* and seven other genes was observed at three more times in the MC and NC groups. Compared with the NC group, *IGFBP5*, *SRY-box containing gene 11*, *phosphatidylethanolamine-binding protein 4*, *keratin 79*, *parkin 2* and *fructosamine-3-kinase* genes were upregulated in the MC group. *FGF5*, *sprouty RTK signaling antagonist 4*, *mesenchymal stem cells*, *PTGS2*, *neuregulin 1* and *TGFα* genes were downregulated in the MC group compared with their expression in the NC group. Other differentially expressed genes between the two libraries included *POU domain, class 2*, *transcription factor 3*; *IGFBP3*, *TIMP3*, *VEGFC*, *G protein-coupled receptor 151* and *SOCS3* (Additional file 1: Table S1).

### GO and KEGG pathway analysis of DEGs

To further investigate the biological function of DEGs, GO analysis was performed through running queries for each DEG against the GO database. Additional file 2: Figure S1 presents these GO functional annotations. Overall, GO terms were assigned to DEGs and classified into three independent ontology categories: (1) 334 terms in biological processes, (2) 209 terms in molecular functions and (3) 284 terms in cellular components. The most important enriched terms are bound up with biological regulation, single-organism processes and cellular processes. These terms were followed by developmental and metabolic processes. The *IGFBP5* and *IGF2* genes in the GO:0005576 term are known to affect mammary development and milk secretion [33, 34], which were downregulated in the MC group. In the cellular component, GO terms with high levels of significance comprised cell parts, organelles and membrane parts (Additional file 2: Figure S1). This organelle is linked with lipid secretor mechanisms of mammary epithelial cells [35]. Additionally, cell and membrane parts have effects on mammary gland involution [36].

Different genes typically cooperate with each other to exercise their biological functions. Pathway-based analyses provide additional insight into the biological functions of genes. Pathway enrichment analysis identifies significantly enriched metabolic pathways or signal transduction pathways related to DEGs. Overall, 22 pathways were significantly enriched (Q value < 0.05) for DEGs (Additional file 3: Figure S2, Additional file 4: Table S2). The prolactin signaling pathways included *FOS*, *ELF5*, *LHβ*, *SOCS3* and *PIK3R3* genes. *IGF2*, *FOS*, *TGFα, PIK3R3,* and *EREG* genes regulate mammary cell growth, apoptosis, proliferation and survival through the TNF and ErbB signaling pathways and proteoglycans [37–39]. KEGG pathway analyses and previous studies [40–44] showed that *TGFα*, *IGFBP5, PIK3R3*, *IGF2*, *ELF5* and *LHβ* play key roles in mammary development and milk secretion.

### Effect of chi-miR-3031 on IGFBP5 expression level

**Fig. 1:**
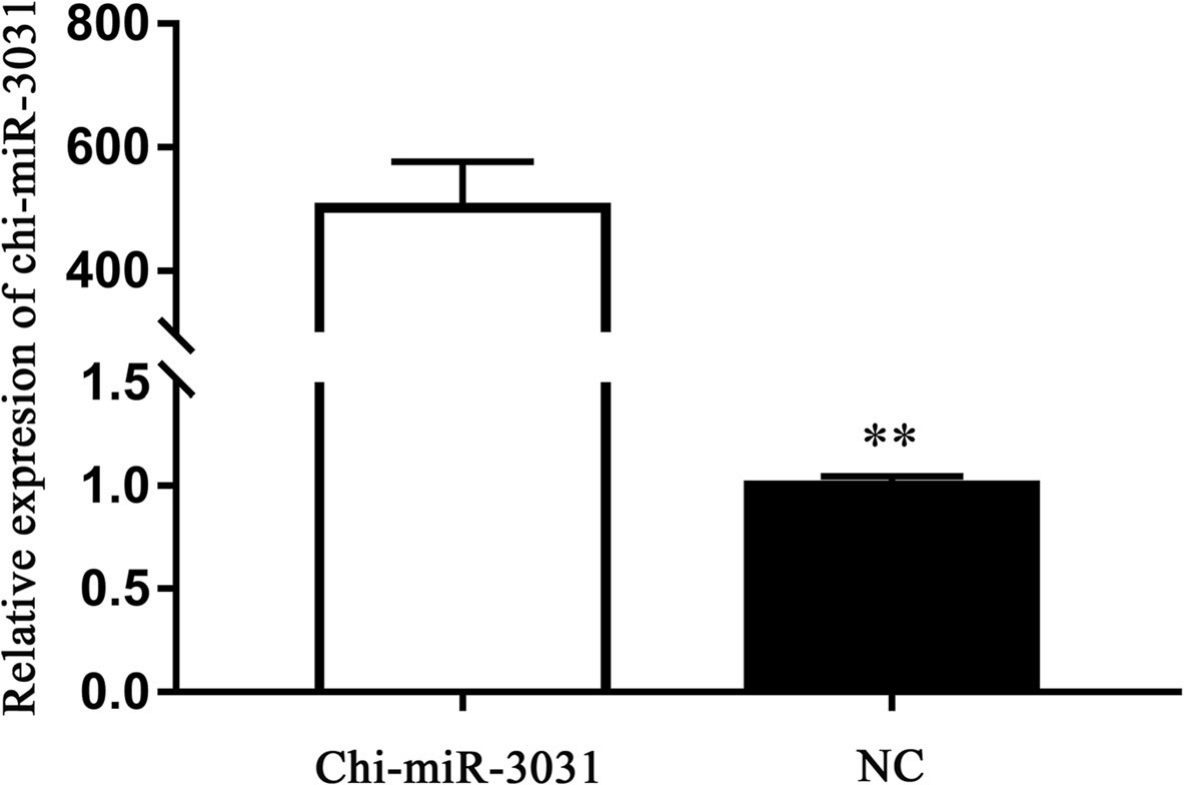
Expression levels of chi-miR-3031 in GMECs transfected with MC and NC. **P < 0.01. NC: Negative control

**Fig. 2:**
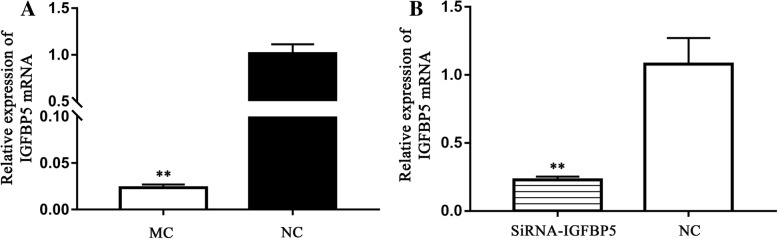
Increasing chi-miR-3031 and siRNA-IGFBP5 levels notably decreased IGFBP5 mRNA expression. **a**: Effect of chi-miR-3031 on IGFBP5 mRNA. **b**: Effect of siRNA-IGFBP5 on IGFBP5 mRNA. *P* < 0.01 is shown as **. MC: Chi-miR-3031 mimic. NC: Negative control

**Fig. 3:**
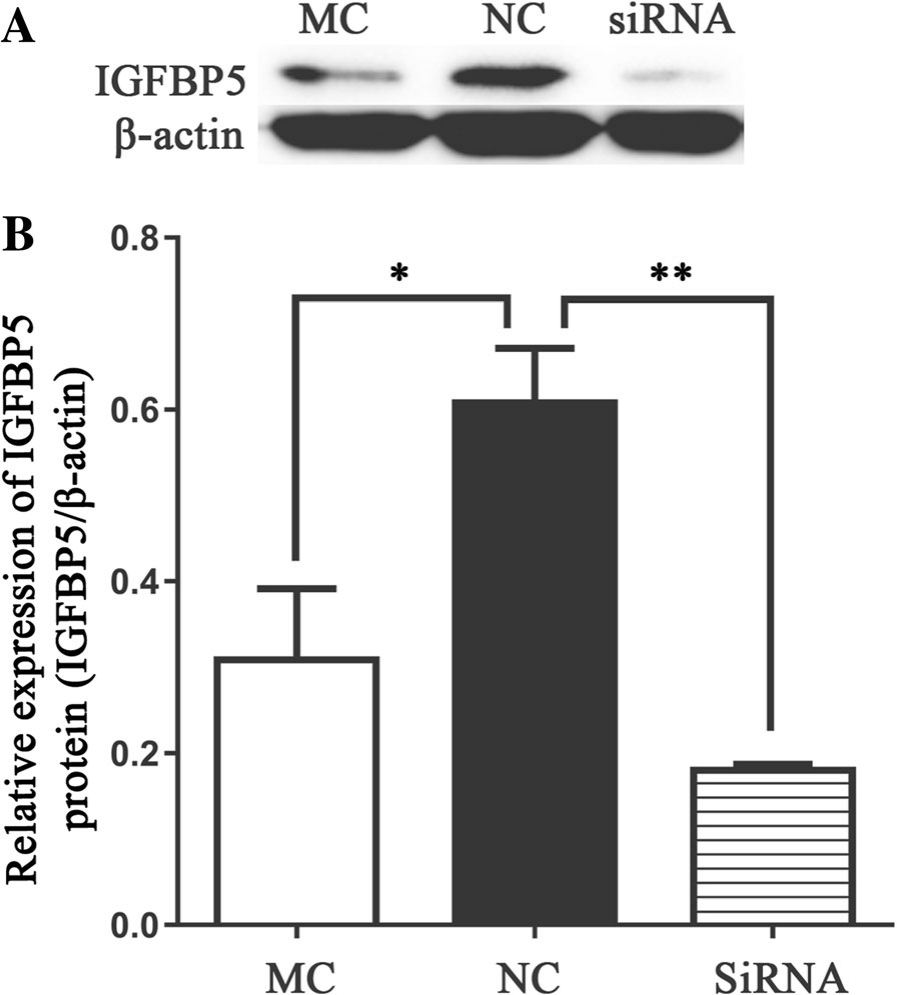
Effects of chi-miR-3031 and siRNA-IGFBP5 on IGFBP5 protein levels. **a**: Western blot analysis results. **b**: Densitometric quantification of western blot results. Protein levels were normalized to β-actin. *P* < 0.05 is shown as *, and *P* < 0.01 is shown as **. MC: Chi-miR-3031 mimic. NC: Negative control

To examine whether chi-miR-3031 can reduce endogenous IGFBP5 expression, GMECs were transfected with MC, siRNA and NC. After 36 h of culture, total RNAs were extracted from the MC, siRNA and NC groups to determine the transfection efficiency of chi-miR-3031 and IGFBP5 mRNA expression. RT-qPCR results showed that the expression level of chi-miR-3031 in the MC group were 500 times higher than that of the NC group (Fig. 1; *P* < 0.01). Additionally, the expression level of *IGFBP5* mRNA in GMECs markedly decreased in the MC group (Fig. 2a; *P* < 0.01), confirming transcriptome sequencing results. Western blot results showed that the expression level of IGFBP5 protein was significantly reduced in the MC group when compared with the NC group (Fig. 3; *P* < 0.05). SiRNA-IGFBP5 significantly reduced the expression levels of IGFBP5 mRNA and protein (Fig. 2b and Fig. 3; *P* < 0.01). These results suggest that chi-miR-3031 plays a negative regulatory role in IGFBP5 expression.

### Chi-miR-3031 and IGFBP5 promote β-casein protein expression

**Fig. 4:**
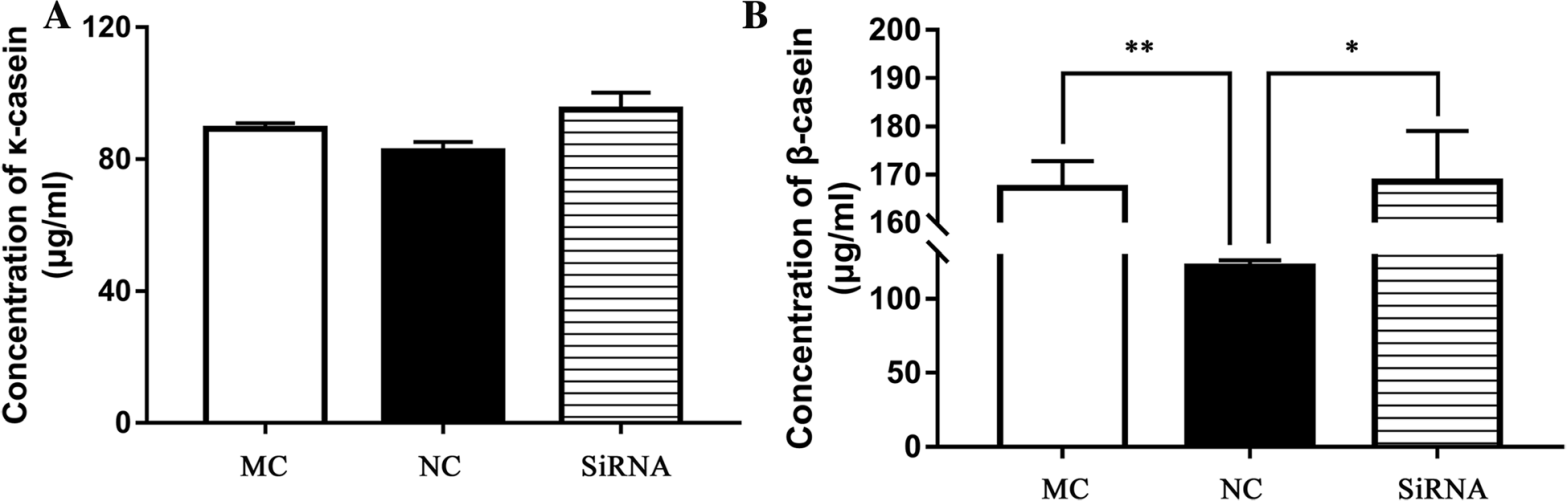
Protein expression levels of κ-casein (**a**) and β-casein (**b**) in GMECs transfected with MC, NC and siRNA-IGFBP5. *P* < 0.05 is shown as *, and *P* < 0.01 is shown as **. MC: Chi-miR-3031 mimic. NC: Negative control

To detect the concentrations of β-casein and κ-casein, GMECs were transfected with NC or MC by direct lysis after 36 h of culture. ELISA results showed that the expression levels of β-casein protein significantly increased in the MC and siRNA-IGFBP5 groups when compared with the NC group (Fig. 4b; *P* < 0.01 or *P* < 0.05). However, the expression levels of κ-casein protein showed no significant differences among the NC, MC and siRNA-IGFBP5 groups (Fig. 4a).

### Chi-miR-3031 induces p-mTOR expression via downregulating IGFBP5

**Fig. 5:**
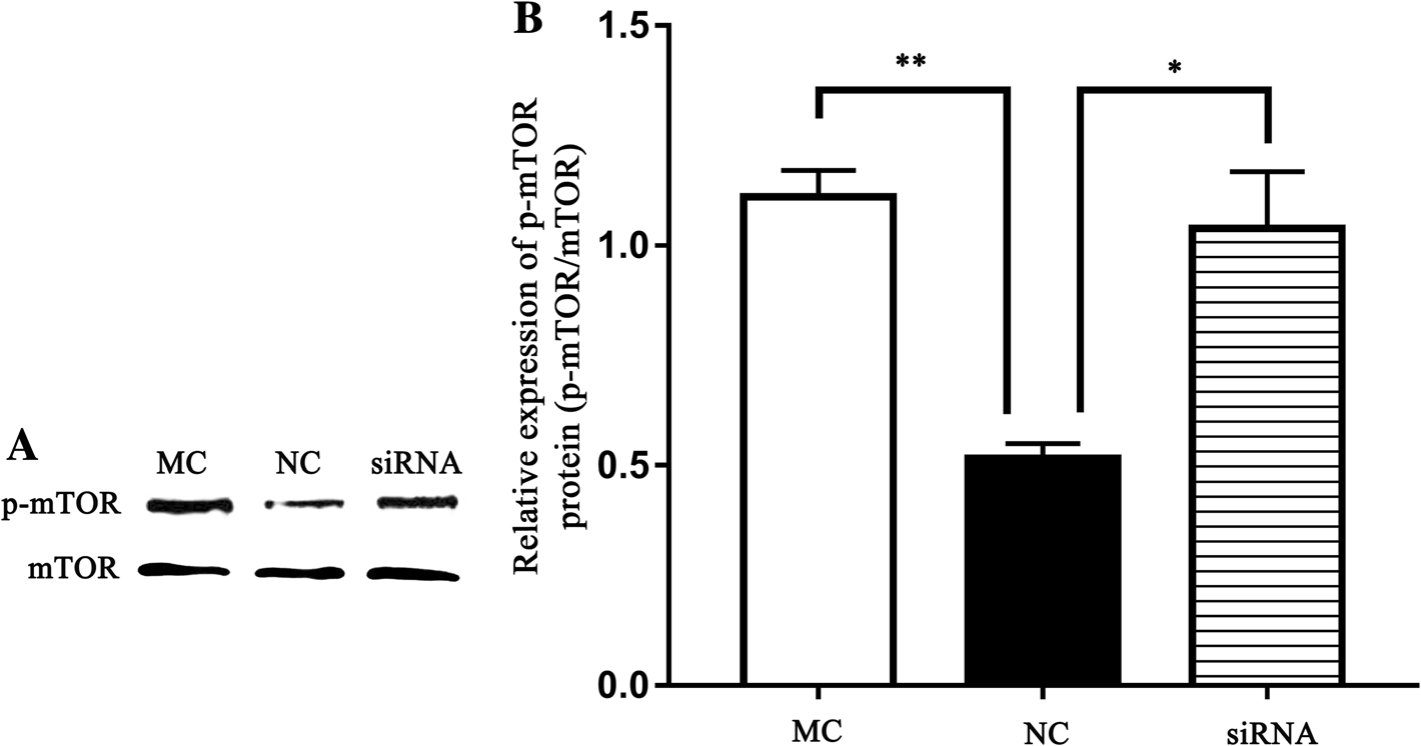
Effects of chi-miR-3031 and siRNA-IGFBP5 on p-mTOR protein levels. **a**: Western blot analysis results. **b**: Densitometric quantification of western blot results. *P* < 0.05 is shown as *, and *P* < 0.01 is shown as **. MC: Chi-miR-3031 mimic. NC: Negative control

Previous studies have suggested that PI3K/AKT-mTOR signaling pathways regulate milk protein synthesis [45]. Thus, this study investigated the expression levels of p-mTOR in GMECs transfected with NC, siRNA or MC. Western blot results showed that chi-miR-3031 and siRNA-IGFBP5 induced the expression of p-mTOR (Fig. 5), suggesting that chi-miRNA-3031 can promote the expression of p-mTOR by downregulating IGFBP5.

## Discussion

MiRNAs play key roles in the development of milk components [46, 47] and mammary glands [48]. However, only a limited number of genes are known to be directly or indirectly regulated by miRNAs in GMECs. Thus, the detection and identification of relevant genes are necessary to determine their function. Our previous studies investigated the differential expression of miRNAs in caprine mammary gland tissues during colostrum and common milk stages using high throughput sequencing and bioinformatics. Herein, we focused on chi-miR-3031 due to its prominent roles in influencing mammary gland development and lactation performance [20]. In MC and NC with transfected GMECs, we selected DEGs that were directly or indirectly regulated by chi-miR-3031. A total of 157 up-regulated and 187 downregulated genes were detected in the MC group compared with the NC group. Singh et al. (2016) showed that SOCS1 and SOCS3 mRNA levels increased by 72 h post-milking compared with levels at 6 h post-milking, additionally, IGF1 mRNA levels increased by 192 h post-milking, whereas IGF2 mRNA decreased by 18 h post-milking compared with 6 h post-milking [49]. In this study, *Fos* and *SOCS3* genes in the prolactin signaling pathway were up-regulated, whereas *ELF5*, *LHβ* and *PIK3R3* genes were down-regulated in the MC group. In cultured mammary gland adipocytes, Prokesch et al. (2014) showed that transient and stable ectopic expression of ELF5 induces the expression of whey acidic protein, a milk component; however, the general adipocyte phenotype remained unaffected, suggesting that additional pioneering factors are necessary [50]. Fifteen members of cancer pathways, including *FOS*, *TGF*α, *FZD8*, *MMP1*, *laminin subunit gamma 2*, *PTGS2*, *laminin subunit beta 3*, *ITGA2*, laminin subunit alpha 3, *VEGFC*, *WNT2B*, *heat shock protein 90 beta family member 1*, *WNT5B*, *FGF5* and *WNT3A,* were up-regulated in the MC group. *WNT10A*, *PIK3R3*, and *VEGFD* were down-regulated. *FZD8*, *plasminogen activator, urokinase receptor*, *ITGA2*, *WNT2B*, *TIMP3*, *WNT5B*, *FGF5*, heparanase, *HBEGF* and *WNT3A* were up-regulated in the proteoglycan pathways in cancer, whereas *WNT10A*, *PIK3R3*, *IGF2* were down-regulated. Nine genes, including *Fos*, *FGF5*, *ITGA2*, *FZD8*, *PIK3R3*, *WNT2B*, *WNT3A*, *WNT5B* and *WNT10A,* are involved in two or three pathways. Thus, the differential expression of these genes could play roles in regulating lactation or mammary gland development.

Several authors have showed the impact of miRNAs on mammary gland development or lactation [21, 46, 51]. Recently, Do et al. (2017) suggested that 19 miRNAs are differentially expressed across lactation in remarkable and time-dependent manners [52]. Additionally, miR-221/222 cluster, miR-18a and transcription factors homeobox A7, and NOTCH 3 and 4 have important roles in regulating lactose. MiR-186, miR-148a and miR-200a are remarkably associated with milk yield and are likely the most important miRNAs for component traits [53]. MiR-130a overexpression decreased *FABP3*, *PPARG*, *FATP1* and *PLIN2* mRNA expression levels, whereas miR-130a downregulation increased *PPARG*, *C/EBP beta*, *C/EBP alpha*, *PLIN2*, *FABP3* and *FATP1* mRNA expression levels [19]. In this study, results from transcriptome sequencing, real-time PCR and western blot showed that chi-miR-3031 can suppress IGFBP5 mRNA and protein expression levels. However, β-casein expression levels significantly increased in the MC and siRNA-IGFBP5 groups. IGFBP5 is an important member of the IGFBP family and has high affinity for IGF [54]. IGFs act as key upstream activators of PI3K/AKT-mTOR signaling pathways that regulate milk protein synthesis [29, 31]. We observed that the down-regulation of IGFBP5 can activate the phosphorylation of mTOR at the Ser2448 site in GMECs transfected with MC and siRNA-IGFBP5. Previous findings have suggested that the activation of p-mTOR implies activation of the PI3K pathway [55]. We speculated that chi-miR-3031 can activate the PI3K/AKT-mTOR pathway and increase β-casein expression by down-regulating IGFBP5. However, κ-casein was not affected by chi-miR-3031.

## Conclusion

In this study, 157 upregulated and 187 downregulated genes were detected by transcriptome sequencing in the MC group. KEGG pathway analyses and previous studies showed that *TGFα*, *PIK3R3*, *IGF2*, *IGFBP5*, *ELF5* and *LHβ* play key roles in mammary development and milk secretion. Thus, the differential expression of these genes can take part in the regulation of lactation or mammary gland development. Additionally, chi-miR-3031 can activate the PI3K/AKT-mTOR pathway and improve the expression of β-casein by down-regulating IGFBP5. Therefore, we conclude that chi-miR-3031 plays key roles in regulating β-casein synthesis in GMECs.

## Methods

### Animals and cell culture

Three Guanzhong dairy goats (3 years old, female) at peak lactation (90 days postpartum) periods were selected from a local farm in the Northwest A&F University of China. These Guanzhong dairy goats are research animals. Half-hour before the operation, these animals were anesthetized by intramuscular injection of 150 mg phenobarbital sodium. In the middle part of mammary gland, open the wound of 1 cm with a scalpel, remove about 1 cm^3^ of mammary gland tissue, then sew and sterilize the wound. The special person kept the experimental animals, and after 1 week, the surgical line was removed, and all the animals recovered. Mammary gland tissues for culturing GMECs were stored in PBS containing streptomycin (100 mg/mL) and penicillin (100 U/mL). GMECs were cultured on the basis of a previous study [56].

### Transfection and total RNA extraction

In accordance with the Lipofectamine RNAiMAX Reagent (Invitrogen, Carlsbad, CA, USA) protocol, GMECs were transfected with MC, NC and siRNA-IGFBP5 at final concentrations of 100 nmol/L. The MC, siRNA and NC were chemically synthesized and purified by Shanghai GenePharma (Shanghai, China). GMECs were cultured for another 36 h after transfection, and TRIzol reagent was used to extract total RNA. The quality of total RNA was detected using an Agilent 2100 Bioanalyzer (Agilent Technologies, Palo Alto, CA, USA). The RIN values of the MC group were 9.2, 9.0 and 8.8, and those of the NC group were 9.3, 9.1 and 8.9. Additional file 5: Table S3 provides the sequences of MC, NC and siRNA.

### Transcriptome sequencing

#### Library construction and sequencing

For each independent experiment, three wells of a 6-well plate were mixed to extract the total RNAs to construct the MC group. The NC group was constructed in the same way. The total RNAs for each group were homogenized to maintain the same concentrations. Sequencing libraries were constructed with NEBNext Ultra RNA Library Prep Kit for Illumina (NEB, Beijing, China). Two MC groups were independently sequenced, respectively. The NC group was sequenced in the same way. Then, in accordance with instructions for use, libraries with different indices were loaded on an Illumina HiSeq instrument (Illumina, San Diego, CA, USA). Sequencing data is processed and analyzed by Personalbio (Shanghai, China).

#### Mapping reads on goat reference genome and analysis of gene expression

After removal of the 3ˊadaptor sequence, low-quality and redundant reads were removed using Cutadapt (version 1.9.1) and clean data were aligned to caprine genome via Hisat2 software (v2.0.1). Caprine genome sequences and gene model annotation files were downloaded from the NCBI genome website (GCF_001704415.1_ASM170441v1).

The level of gene expression was measured by read density, and gene expression calculation was performed with Cuffdiff (v2.2.1), which calculates fragments per kilo bases per million (FPKM) reads as FPKM = total exon reads/mapped reads in millions × exon length in kb. The results from the cuffdiff analysis were further analyzed to identify genes with significant differential expression according to the criteria of fold-changes greater than 1 and *p* values less than 0.05.

#### Gene ontology (GO) and KEGG pathway analysis of DEGs

The DEGs were mapped to GO terms in the Gene Ontology database (http://www.geneontology.org/). Remarkably enriched GO terms in differentially methylated genes were defined by hypergeometric test [57–59]. Pathway-based analyses aid in further understanding gene function [60]. The calculation formula of KEGG (http://en.wikipedia.org/wiki/KEGG) [61] was the same with that of GO analysis. Pathway analysis identified remarkably enriched signal transduction pathways for DEGs [62].

### Reverse transcription and real-time PCR

Table 3 shows the names of miRNA and genes for reverse transcription and real-time PCR. The reverse transcription system contained the following: (1) 800 ng of total RNA, 1 μL of gDNA Eraser, 2 μL of 5 × gDNA Eraser Buffer and RNase-Free dH_2_O mixed to a final volume of 10 μL and incubated at 42 °C for 2 min. (2) 4 μL of 5 × Prime Script Buffer 2, 1 μL of RT Primer Mix, 1 μL of PrimeScript RT Enzyme Mix 1 and RNase-Free dH_2_O mixed to a final volume of 20 μL and incubated at 42 °C for 15 min followed by 85 °C for 5 s using a PrimeScript RT reagent Kit (TaKaRa, Dalian, China). cDNA products were stored at − 20 °C.

**Table 3:** Primer information for RT-qPCR

Name	Primer	Primer sequence (5′ → 3′)	Size (bp)	Tm (°C)
Chi-miR-3031	RT-Primer	GTCGTATCCAGTGCAGGGTCCGAGGTATTCGCACTGGATACGACGGGGCCGA		
	Forward primer	TTATGGGCTGGCTCCCTC	42	60
	Reverse primer	CAGTGCAGGGTCCGAGGT		
*U*_*6*_	Forward primer	CTCGCTTCGGCAGCACA	89	60
	Reverse primer	AACGCTTCACGAATTTGCGT		
*IGFBP5*	Forward primer	GTACCTGCCCAACTGTGACC	116	60
	Reverse primer	CTGGCAGCTTCATCCCATAC		
*β-casein*	Forward primer	ACAGCCTCCCACAAAACATCC	297	60
	Reverse primer	TGAGAAAGGGACAGCACGGA		
*β-actin*	Forward primer	TGACCCAGATCATGTTTGAGA	186	60
	Reverse primer	CAAGGTCCAGACGCAGGAT		

Real-time PCR was performed with a 25 μL volume containing 2 μL of template cDNA, 12.5 μL of SYBR Premix Ex Taq II (TaKaRa, Dalian, China) and 1 μM of primers using the CFX Connect Real-Time PCR Detection System (Bio-Rad, CA, USA). Thermal cycling conditions were 95 °C for 10 min, followed by 40 cycles at 94 °C for 15 s, 60 °C for 30 s and 72 °C for 30 s. Primers are shown in Table 3. The *β-actin* or *U*_*6*_ mRNA levels were used for normalization. Each experiment was independently repeated at least three times, and fold changes were analyzed via the 2^-ΔΔCt^ method.

### Enzyme-linked immunosorbent assay

ELISA was performed to detect the concentration of caseins in cell lysates using goat β-casein and κ-casein kit (Tongwei, Shanghai, China). Briefly, GMECs were transfected with NC or MC after 36 h of culture in six-well plates and then lysed with 200 μL RIPA Lysis Buffer (Bioteke, Beijing, China). According to manufacturer instructions, 50 μL cell lysate or standards were used, and absorbance at 450 nm was measured through an Epoch microplate reader (Biotek, Winooski, USA). Concentrations were calculated after subtracting the standard curve. Kit sensitivity was 1.0 μg/mL, and mean intra- and inter-assay variable coefficient values were less than 15 and 10%, respectively.

### Western blotting

GMECs were lysed in denaturing lysis buffer containing protease inhibitors (RIPA, Bioteke, Beijing, China) for 30 min on ice and centrifuged (12,000×g) for 15 min at 4 °C. Protein concentrations in the lysates were determined by a BCA protein assay kit (Vazyme Biotech, Nanjing, China). Exactly 30 μg of protein were separated on a 12% SDS-PAGE and transferred to a polyvinylidene difluoride membrane that was blocked with 5% nonfat dried milk in Tris-buffered saline containing 0.1% Tween 20 (pH 7.6) for 1 h at 25 °C. Subsequently, the membranes were incubated overnight at 4 °C with a rabbit anti-IGFBP5 polyclonal antibody (1:300, Boster, USA), a rabbit anti-mTOR polyclonal antibody (1:100, Boster, USA), a rabbit anti-p-mTOR polyclonal antibody (1:100, Boster, USA) or a mouse β-actin monoclonal primary antibody (1:1000, Beyotime, Jiangsu, China). Then, the blot was incubated with a horseradish peroxidase-conjugated secondary antibody for 1 h at 25 °C. The quantification of protein was detected through the Quantity One program (Bio-Rad, California, USA).

### Statistical analysis

The one-way ANOVA of SPSS 16.0 software package was used to analyze data. Means of multiple comparisons was analyzed by least significant difference (LSD). Data were presented as means ± standard deviation of three independent experiments. *P* < 0.05 or *P* < 0.01 was considered significant.

## Additional files

Additional file 1: **Table S1.** The up and down-regulated genes in the chi-miR-3031 mimics VS. negative control groups. (XLSX 46 kb)

Additional file 2: **Figure S1.** GO analysis results based on biological process, cellular component and molecular function. (TIF 5910 kb)

Additional file 3: **Figure S2.** KEGG pathway analysis for DEGs. (TIF 2340 kb)

Additional file 4: **Table S2.** Pathway annotations of differentially expressed genes. (XLSX 13 kb)

Additional file 5: **Table S3.** Sequence information of chi-miR-3031 mimics, mimic negative control and siRNA-IGFBP5. (DOCX 16 kb)

## Abbreviations

AKT: Protein kinase B
C/EBP: CCAAT/enhancer binding protein
CAM: Cell adhesion molecule
DEGs: Differentially expressed genes
ELF5: E74-like factor 5
ELISA: Enzyme linked immunosorbent assay
EREG: Epiregulin
FABP3: Fatty acid binding protein 3
FATP1: Fatty acid transport protein
FGF5: Fibroblast growth factor 5
FOS: FBJ osteosarcoma oncogene
FPKM: Fragments Per Kilo base per Million reads
FZD8: Frizzled class receptor 8
GMECs: Goat mammary epithelial cells
GO: Gene ontology
HB-EGF: Heparin-binding EGF-like growth factor
IGF: Insulin-like growth factor
IGFBPs: IGF-binding proteins
ITGA2: Integrin alpha 2
KEGG: Kyoto encyclopedia of genes and genomes
LHβ: Luteinizing hormone beta subunit
miRNAs: MicroRNAs
MMP1: Metalloproteinase-1
mTOR: Phosphorylation of mechanistic target of rapamycin
PBS: Phosphate-buffered saline
PI3K: Phosphoinositide 3-kinase
PIK3R3: Phosphatidylinositol 3-kinase regulatory subunit gamma
PLIN2: Perilipin 2
p-mTOR: Phosphorylated mTOR
PPARG: Peroxisome proliferator activated receptor gamma
PTGS2: Prostaglandin-endoperoxide-synthase-1 gene
RNA-Seq: High-throughput sequencing of RNA
SOCS3: Suppressor of cytokine signaling 3
STAT: Signal transducer and activator of transcription
TAG: Triacylglycerol
TGFα: Transforming growth factor alpha
TIMP3: Tissue inhibitor of metalloproteinases-3
VEGFC: Vascular endothelial growth factor C
WNT2B: Wnt family member 2B
β-casein: Beta-casein

## References

[1] Zhang R, Ma HM, Gao Y, Wu YJ, Qiao YM, Geng A, Cai CG, Han YY, Zeng YA, Liu XL, Ge GX (2018). Th-POK regulates mammary gland lactation through mTOR-SREBP pathway. PLoS Genet.

[2] Saipin N, Noophun J, Chumyim P, Rungsiwiwut R (2018). Goat milk: non-invasive source for mammary epithelial cell isolation and in vitro culture. Anatomia Histologia Embryologia.

[3] Yang Y, Fang X, Yang R, Yu H, Jiang P, Sun B, Zhao Z (2018). MiR-152 regulates apoptosis and triglyceride production in MECs via targeting ACAA2 and HSD17B12 genes. Sci Rep.

[4] Huppertz, T. 2013. Chemistry of the caseins. Pages 135–160 in Advanced dairy chemistry. Vol. 1A: Proteins: Basic Aspects. 4th ed. P. L. H. McSweeney and P. F. Fox, ed. Springer Science+Business Media, New York, NY.

[5] Li H, Ruan J, Durbin R (2008). Mapping short DNA sequencing reads and calling variants using mapping quality scores. Genome Res.

[6] Cloonan N, Grimmond SM (2008). Transcriptome content and dynamics at single-nucleotide resolution. Genome Biol.

[7] Lister R, O'Malley RC, Tonti-Filippini J, Gregory BD, Berry CC, Millar AH, Ecker JR (2008). Highly integrated single-base resolution maps of the epigenome in Arabidopsis. Cell.

[8] Marioni JC, Mason CE, Mane SM, Stephens M, Gilad Y (2008). RNA-seq: an assessment of technical reproducibility and comparison with gene expression arrays. Genome Res.

[9] Grabherr MG, Haas BJ, Yassour M, Levin JZ, Thompson DA, Amit I, Adiconis X, Fan L, Raychowdhury R, Zeng Q (2011). Full-length transcriptome assembly from RNA-Seq data without a reference genome. Nat Biotechnol.

[10] Suarez-Vega A, Gutierrez-Gil B, Klopp C, Tosser-Klopp G, Arranz JJ (2017). Variant discovery in the sheep milk transcriptome using RNA sequencing. BMC Genomics.

[11] Wanyonyi SS, Kumar A, Du Preez R, Lefevre C, Nicholas KR (2017). Transcriptome analysis of mammary epithelial cell gene expression reveals novel roles of the extracellular matrix. Biochem Biophys Rep.

[12] Croce CM, Calin GA (2005). miRNAs, cancer, and stem cell division. Cell.

[13] Bartel DP (2009). MicroRNA target recognition and regulatory functions. Cell.

[14] Shiah SG, Hsiao JR, Chang WM, Chen YW, Jin YT, Wong TY, Huang JS, Tsai ST, Hsu YM, Chou ST (2014). Downregulated miR-329 and miR-410 promote the proliferation and invasion of oral squamous cell carcinoma by targeting Wnt-7b. Cancer Res.

[15] Ambros V (2003). MicroRNA pathways in flies and worms: growth, death, fat, stress, and timing. Cell.

[16] An X, Song Y, Hou J, Li G, Zhao H, Wang J, Cao B (2016). Identification and profiling of microRNAs in the ovaries of polytocous and monotocous goats during estrus. Theriogenology.

[17] Li HM, Wang CM, Li QZ, Gao XJ (2012). MiR-15a decreases bovine mammary epithelial cell viability and lactation and regulates growth hormone receptor expression. Molecules.

[18] Cui YJ, Sun X, Jin LF, Yu GP, Li QZ, Gao XJ, Ao JX, Wang CM (2017). MiR-139 suppresses beta-casein synthesis and proliferation in bovine mammary epithelial cells by targeting the GHR and IGF1R signaling pathways. BMC Vet Res.

[19] Yang WC, Guo WL, Zan LS, Wang YN, Tang KQ (2017). Bta-miR-130a regulates the biosynthesis of bovine milk fat by targeting peroxisome proliferator-activated receptor gamma. J Anim Sci.

[20] Hou JX, An XP, Song YX, Cao BY, Yang HP, Zhang Z, Shen WZ, Li YP (2017). Detection and comparison of microRNAs in the caprine mammary gland tissues of colostrum and common milk stages. BMC Genet.

[21] Chu M, Zhao Y, Yu S, Hao Y, Zhang P, Feng Y, Zhang H, Ma D, Liu J, Cheng M (2017). MiR-15b negatively correlates with lipid metabolism in mammary epithelial cells. Am J Physiol Cell Physiol.

[22] Wang H, Shi H, Luo J, Yi Y, Yao D, Zhang X, Ma G, Loor JJ (2017). MiR-145 regulates lipogenesis in goat mammary cells via targeting INSIG1 and epigenetic regulation of lipid-related genes. J Cell Physiol.

[23] Hwa V, Oh Y, Rosenfeld RG (1999). Insulin-like growth factor binding proteins: a proposed superfamily. Acta Paediatr.

[24] Zhang H, Shi Y, He M (2017). Molecular identification of an insulin growth factor binding protein (IGFBP) and its potential role in an insulin-like peptide system of the pearl oyster, Pinctada fucata. Comp Biochem Physiol B Biochem Mol Biol.

[25] Murphy M, Pykett MJ, Harnish P, Zang KD, George DL (1993). Identification and characterization of genes differentially expressed in meningiomas. Cell Growth Differ.

[26] Denley A, Cosgrove LJ, Booker GW, Wallace JC, Forbes BE (2005). Molecular interactions of the IGF system. Cytokine Growth Factor Rev.

[27] Pera EM, Wessely O, Li SY, De Robertis EM (2001). Neural and head induction by insulin-like growth factor signals. Dev Cell.

[28] Salih DAM, Tripathi G, Holding C, Szestak TAM, Gonzalez MI, Carter EJ, Cobb LJ, Eisemann JE, Pell JM (2004). Insulin-like growth factor-binding protein 5 (Igfbp5) compromises survival, growth, muscle development, and fertility in mice. Proc Natl Acad Sci U S A.

[29] Ma JC, Sawai H, Matsuo Y, Ochi N, Yasuda A, Takahashi H, Wakasugi T, Funahashi H, Sato M, Takeyama H (2010). IGF-1 mediates PTEN suppression and enhances cell invasion and proliferation via activation of the IGF-1/PI3K/Akt signaling pathway in pancreatic Cancer cells. J Surg Res.

[30] Bionaz M, Loor JJ (2008). Gene networks driving bovine milk fat synthesis during the lactation cycle. BMC Genomics.

[31] Sobolewska A, Gajewska M, Zarzyńska J, Gajkowska B, Motyl T (2009). IGF-I, EGF, and sex steroids regulate autophagy in bovine mammary epithelial cells via the mTOR pathway. Eur J Cell Biol.

[32] Murney R, Stelwagen K, Wheeler TT, Margerison JK, Singh K (2015). The effects of milking frequency on insulin-like growth factor I signaling within the mammary gland of dairy cows. J Dairy Sci.

[33] Bomfim GF, Merighe GKF, de Oliveira SA, Negrao JA (2018). Effect of acute stressors, adrenocorticotropic hormone administration, and cortisol release on milk yield, the expression of key genes, proliferation, and apoptosis in goat mammary epithelial cells. J Dairy Sci.

[34] De Silva D, Kunasegaran K, Ghosh S, Pietersen AM (2015). Transcriptome analysis of the hormone-sensing cells in mammary epithelial reveals dynamic changes in early pregnancy. BMC Dev Biol.

[35] Ghosal D, Shappell NW, Keenan TW (1994). Endoplasmic reticulum lumenal proteins of rat mammary gland-potential involvement in lipid droplet assembly during lactation. Biochim Biophys Acta.

[36] Suárez-Vega A, Gutiérrez-Gil B, Klopp C, Robert-Granie C, Tosser-Klopp G, Arranz JJ (2015). Characterization and comparative analysis of the milk transcriptome in two dairy sheep breeds using RNA sequencing. Sci Rep.

[37] Kosciuczuk EM, Lisowski P, Jarczak J, Majewska A, Rzewuska M, Zwierzchowski L, Bagnicka E (2017). Transcriptome profiling of staphylococci-infected cow mammary gland parenchyma. BMC Vet Res.

[38] Bade LK, Goldberg JE, Dehut HA, Hall MK, Schwertfeger KL (2011). Mammary tumorigenesis induced by fibroblast growth factor receptor 1 requires activation of the epidermal growth factor receptor. Journal of Cell Sci.

[39] Williams MM, Vaught DB, Morrison-Joly M, Hicks DJ, Sanchez V, Owens P, Rahman B, Elion DL, Balko JM, Cook RS (2017). ErbB3 drives mammary epithelial survival and differentiation during pregnancy and lactation. Breast Cancer Res.

[40] O'Leary KA, Jallow F, Rugowski DE, Sullivan R, Sinkevicius KW, Greene GL, Schuler LA (2013). Prolactin activates ER alpha in the absence of ligand in female mammary development and carcinogenesis in vivo. Endocrinology.

[41] Stahel P, Kim JJ, Cieslar SRL, Warrington JM, Xiao CT, Cant JP (2017). Supranutritional selenium intake from enriched milk casein impairs hepatic insulin sensitivity via attenuated IRS/PI3K/AKT signaling and decreased PGC-1 alpha expression in male Sprague-Dawley rats. J Nutr Biochem.

[42] Chean J, Chen CJ, Shively JE (2017). ETS transcription factor ELF5 induces lumen formation in a 3D model of mammary morphogenesis and its expression is inhibited by Jak2 inhibitor TG101348. Exp Cell Res.

[43] Gao YH, Jiang JP, Yang SH, Hou YL, Liu GE, Zhang SG, Zhang Q, Sun DX (2017). CNV discovery for milk composition traits in dairy cattle using whole genome resequencing. BMC Genomics.

[44] Brozos CN, Kiossis E, Fthenakis GC, Tsousis G, Boscos C (2007). Supplementation of lactating ewes with a glucogenic preparation or beta-carotene in mid- to late lactation on subsequent milk yield and luteinizing hormone secretion. Can J Anim Sci.

[45] Burgos SA, Cant JP (2010). IGF-1 stimulates protein synthesis by enhanced signaling through mTORC1 in bovine mammary epithelial cells. Domest Anim Endoc.

[46] Wang H, Luo J, He QY, Yao DW, Wu J, Loor JJ (2017). miR-26b promoter analysis reveals regulatory mechanisms by lipid-related transcription factors in goat mammary epithelial cells. J Dairy Sci.

[47] Tang KQ, Wang YN, Zan LS, Yang WC (2017). miR-27a controls triacylglycerol synthesis in bovine mammary epithelial cells by targeting peroxisome proliferator-activated receptor gamma. J Dairy Sci.

[48] Sandhu GK, Milevskiy MJG, Wesley W, Shewan AM, Brown MA (2016). Non-coding RNAs in mammary gland development and disease. Adv Exp Med Biol.

[49] Singh K, Vetharaniam I, Dobson JM, Prewitz M, Oden K, Murney R, Swanson KM, Mcdonald R, Henderson HV, Stelwagen K (2016). Cell survival signaling in the bovine mammary gland during the transition from lactation to involution. J Dairy Sci.

[50] Prokesch A, Smorlesi A, Perugini J, Manieri M, Ciarmela P, Mondini E, Trajanoski Z, Kristiansen K, Giordano A, Bogner-Strauss JG (2014). Molecular aspects of adipoepithelial transdifferentiation in mouse mammary gland. Stem Cells.

[51] Zhen L, Liu H, Jin X, Lijan L, Liu J (2012). Expression profiles of microRNAs from lactating and non-lactating bovine mammary glands and identification of miRNA related to lactation. BMC Genomics.

[52] Do DN, Li R, Dudemaine PL, Ibeaghaawemu EM (2017). MicroRNA roles in signalling during lactation: an insight from differential expression, time course and pathway analyses of deep sequence data. Sci Rep.

[53] Do DN, Dudemaine PL, Li R, Ibeagha-Awemu EM (2017). Co-expression network and pathway analyses reveal important modules of miRNAs regulating milk yield and component traits. Int J Mol Sci.

[54] Mcqueeney K, Dealy CN (2001). Roles of insulin-like growth factor-I (IGF-I) and IGF-I binding protein-2 (IGFBP2) and −5 (IGFBP5) in developing chick limbs. Growth Hormon IGF Res.

[55] Altomare DA, Wang HQ, Skele KL, De Rienzo A, Klein-Szanto AJ, Godwin AK, Testa JR (2004). AKT and mTOR phosphorylation is frequently detected in ovarian cancer and can be targeted to disrupt ovarian tumor cell growth. Oncogene.

[56] Chen Z, Luo J, Sun S, Cao D, Shi H, Loor JJ (2017). miR-148a and miR-17-5p synergistically regulate milk TAG synthesis via PPARGC1A and PPARA in goat mammary epithelial cells. RNA Biol.

[57] Harris MA. The gene ontology (GO) database and informatics resource: WCB/McGraw-Hill; 2004. 10.1093/nar/gkh036 PMC308770 14681407

[58] Drabkin HJ, Blake JA, Database MGI (2012). Manual gene ontology annotation workflow at the mouse genome informatics database. Database (Oxford).

[59] Benjamini Y, Yekutieli D (2001). The control of the false discovery rate in multiple testing under dependency. Ann Stat.

[60] Kanehisa M, Araki M, Goto S, Hattori M, Hirakawa M, Itoh M, Katayama T, Kawashima S, Okuda S (2008). Tokimatsu T. KEGG for linking genomes to life and the environment. Nucleic Acids Res.

[61] Ogata H, Goto S, Sato K, Fujibuchi W, Bono H, Kanehisa MKEGG (2000). Kyoto encyclopedia of genes and genomes. Nucleic Acids Res.

[62] Kanehisa M, Goto S, Sato Y, Furumichi M, Mao T (2012). KEGG for integration and interpretation of large-scale molecular data sets. Nucleic Acids Res.

