# Supplementary figures and images for: Chi-miR-3031 regulates beta-casein via the PI3K/AKT-mTOR signaling pathway in goat mammary epithelial cells (GMECs)
Source: BMC Vet Res. 2018 Nov 27;14:369. doi: 10.1186/s12917-018-1695-6 (PMC6258393; doi:10.1186/s12917-018-1695-6)

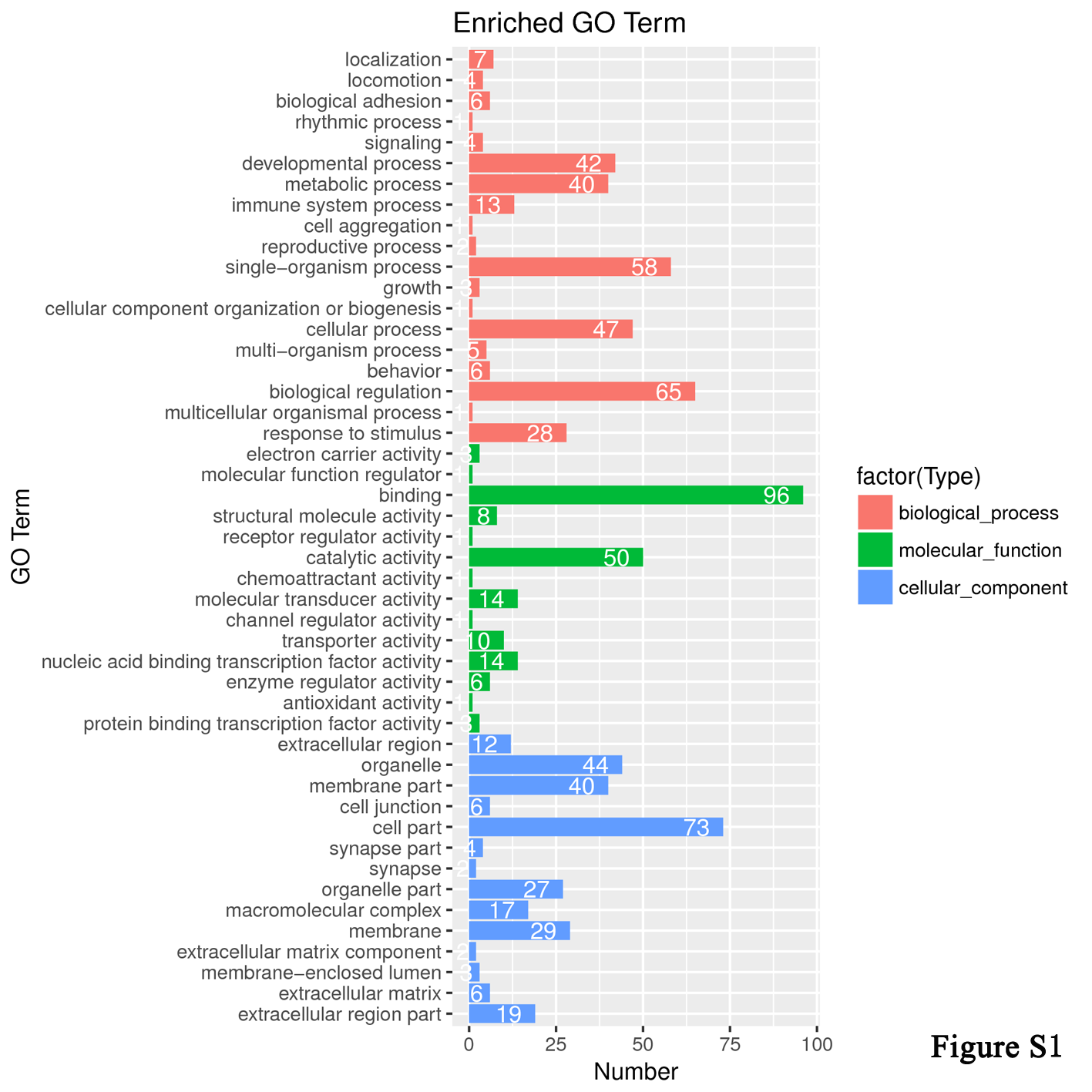

Supplement: Supplementary file 2 — Figure S1. GO analysis results based on biological process, cellular component and molecular function. (TIF 5910 kb) [file 12917_2018_1695_MOESM2_ESM.tif]

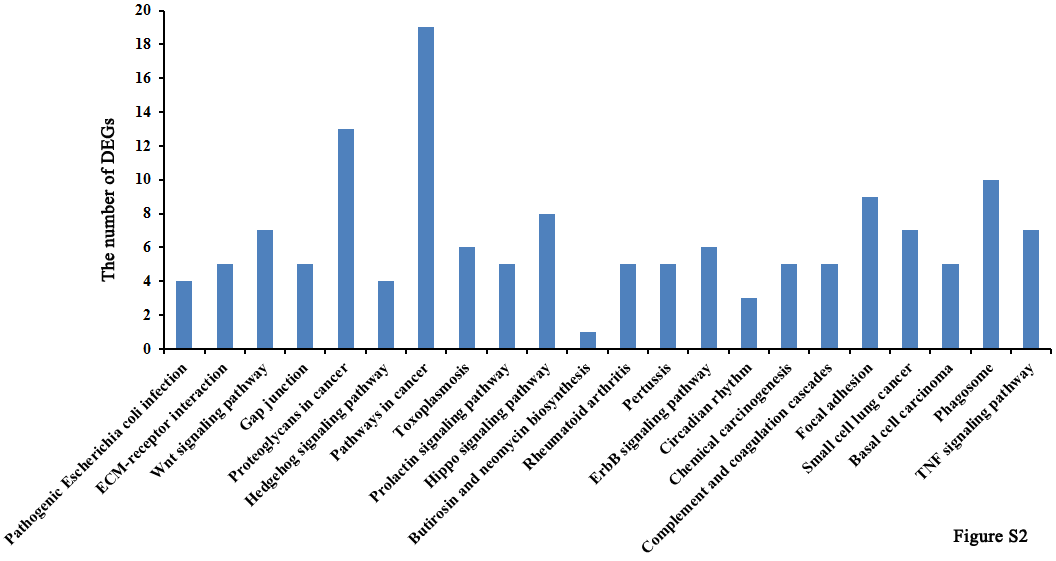

Supplement: Supplementary file 3 — Figure S2. KEGG pathway analysis for DEGs. (TIF 2340 kb) [file 12917_2018_1695_MOESM3_ESM.tif]
