# Supplementary material for: Chi-miR-3031 regulates beta-casein via the PI3K/AKT-mTOR signaling pathway in goat mammary epithelial cells (GMECs)
Source: BMC Vet Res. 2018 Nov 27;14:369. doi: 10.1186/s12917-018-1695-6 (PMC6258393; doi:10.1186/s12917-018-1695-6)
Supplement: Supplementary file 5 — Table S3. Sequence information of chi-miR-3031 mimics, mimic negative control and siRNA-IGFBP5. (DOCX 16 kb) [file 12917_2018_1695_MOESM5_ESM.docx]

**Supplementary information**

**Table S3** Sequence information of chi-miR-3031 mimics, mimic negative control and siRNA-IGFBP5

| Name | Sequence (5'→3') |
| --- | --- |
| Chi-miR-3031 mimic | *GGGCUGGCUCCCUCGGCCCC  GGCCGAGGGAGCCAGCCCUU |
| Mimic negative control | UUCUCCGAACGUGUCACGUTT  ACGUGACACGUUCGGAGAATT |
| SiRNA-IGFBP5 | CCUACUCGCCCAAGAUCUUTT  AAGAUCUUGGGCGAGUAGGTT |

Note: Asterisk shows the sequence of chi-miR-3031
